# Benzyl alcohol oxidation with Pd-Zn/TiO_2_: computational and experimental studies

**DOI:** 10.1080/14686996.2019.1598237

**Published:** 2019-04-25

**Authors:** Ewa Nowicka, Sultan Althahban, Tom D. Leah, Greg Shaw, David Morgan, Christopher J. Kiely, Alberto Roldan, Graham J. Hutchings

**Affiliations:** a Cardiff Catalysis Institute, School of Chemistry, Cardiff University, Cardiff, UK; b Department of Materials Science and Engineering, Lehigh University, Bethlehem, PA, USA; c Department of Mechanical Engineering, Jazan University, Jazan, Saudi Arabia

**Keywords:** Pd–Zn catalyst, Pd–Zn alloys, benzyl alcohol oxidation, Pd–Zn surface, adsorption energy, 60 New topics / Others, 205 Catalyst / Photocatalyst / Photosynthesis

## Abstract

Pd–Zn/TiO_2_ catalysts containing 1 wt% total metal loading, but with different Pd to Zn ratios, were prepared using a modified impregnation method and tested in the solvent-free aerobic oxidation of benzyl alcohol. The catalyst with the higher Pd content exhibited an enhanced activity for benzyl alcohol oxidation. However, the selectivity to benzaldehyde was significantly improved with increasing presence of Zn. The effect of reduction temperature on catalyst activity was investigated for the catalyst having a Pd to Zn metal molar ratio of 9:1. It was found that lower reduction temperature leads to the formation of PdZn nanoparticles with a wide particle size distribution. In contrast, smaller PdZn particles were formed upon catalyst reduction at higher temperatures. Computational studies were performed to compare the adsorption energies of benzyl alcohol and the reaction products (benzaldehyde and toluene) on PdZn surfaces to understand the oxidation mechanism and further explain the correlation between the catalyst composition and its activity.

## Graphical Abstract

**Figure:**
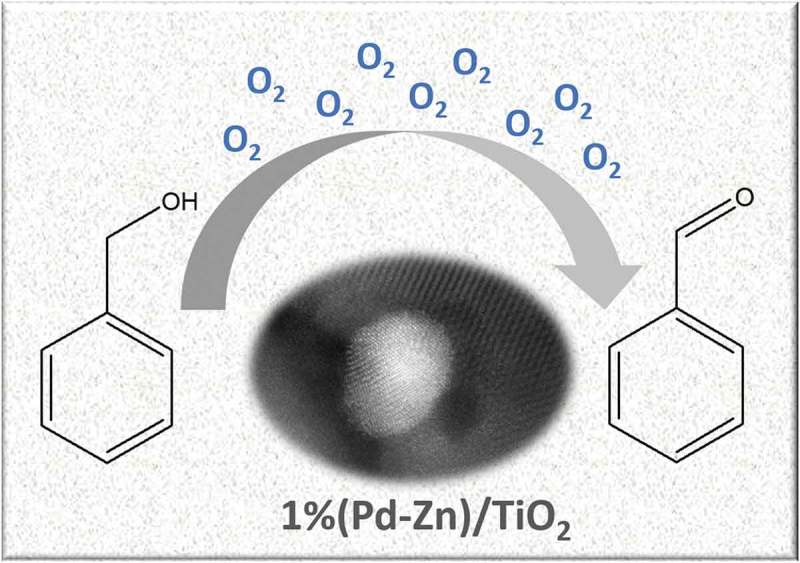

## Introduction

Oxidation is a key reaction in organic synthesis and industrial processes. The products of the oxidation of alcohols, such as aldehydes, are valuable both as intermediates and final components to the pharmaceutical and perfume industries [1]. Numerous methods have been developed for promoting a wide variety of oxidation transformations including the use of stoichiometric and powerful oxidants [2]. However, many of these reactions are unselective, meaning that a number of undesirable side products are formed, which increase the cost of the process because of the subsequent need for purification steps. Therefore, a method to selectively oxidise alcohols with high activity using a benign oxidant, (e.g. molecular oxygen) is highly desirable. Molecular oxygen in combination with a suitable heterogeneous catalyst offers a possible route to perform such reactions in a more efficient and environment-friendly way [3]. Amongst the many catalytic systems tested to date, supported metal nanoparticle catalysts combined with molecular oxygen offers great potential in terms of general environmental friendliness.

The oxidation of benzyl alcohol to benzaldehyde has been one of the most studied systems for selective oxidation of alcohols. This is due to its high reactivity, the limited number of products in the oxidation reaction and relatively well-known pathways for their formation. It has been generally accepted that benzaldehyde is obtained by the oxidation of benzyl alcohol in the presence of an oxidant and can undergo further oxidation to benzoic acid, which can in turn form benzyl benzoate (Equation 1; Ph stands for phenyl group) [4]. Toluene, considered as a by-product of this reaction, is believed to be formed in the disproportionation of benzyl alcohol (Equation 2) [5].

(1) $$PhCH_{2}O\overset{\;}{\to }PhCHO$$

(2) $$2PhCH_{2}OH\to PhCH_{3}+PhCHO$$

A number of monometallic supported nanoparticles have been reported to be active for the oxidation of benzyl alcohol. Mori et al. were amongst the first to report that palladium nanoparticles supported on hydroxyapatite successfully converted benzyl alcohol to benzaldehyde in the presence of molecular oxygen [6]. Further studies showed that although catalytic activity was related to the metal loading, the type of support used in the catalyst preparation determined reaction selectivity. Amongst the many different types of support studied in combination with Pd nanoparticles, Al_2_O_3_, SiO_2_, C and TiO_2_ were the most common [7].

Gold, along with silver and copper, are becoming much more widely studied metals in the selective oxidation of alcohols to aldehydes [8]. Here again a range of supports have been tested in combination with Au nanoparticles; including CeO_2_, Fe_2_O_3_, SiO_2_, TiO_2_ and MgO [9]. In common with the case of supported Pd, it was noticed that selectivity was strongly dependant on support identity with basic supports facilitating easier formation of benzaldehyde.

The alloying of two metals has become a popular technique to improve product selectivity, enhance reaction rate and suppress catalyst deactivation. Often it allows one to conduct experiments at lower reaction temperature, use less catalyst and decrease total metal loading [10]. It is generally considered that the properties of a bimetallic catalyst become changed from their monometallic counterparts due to electronic and/or geometric synergistic effects. Synthesis of bimetallic catalysts for benzyl alcohol oxidation has been mostly focused on Au, Pd, Pt and Ag-based catalysts. Amongst all these bimetallic catalysts, 1% Au-Pd/TiO_2_ deserves special attention; they have been widely studied in the last two decades and generated significant interest especially in the oxidation of alcohols [11]. This is because Au and Pd can form a continuous solid solution over the whole gold/palladium composition range and the addition of the second metal can alter the electronic and geometrical properties of the synthesized particles, positively affecting catalytic activity, catalyst stability and the distribution of products [12]. The preparation of bimetallic nanoalloys plays a very important role and determines final structure, and hence catalytic properties displayed by these materials. Therefore, it is crucial to carefully choose which synthesis method should be used to obtain a bimetallic material with the desired structure and property combination.

One of the main parameters that can be controlled by choosing the appropriate method of catalyst preparation is the size distribution of the resultant nanoparticles. Sankar et al. compared modified impregnation with traditional impregnation and sol immobilization methods and showed that by adding excess of Cl^−^ to the precursor mixture, better control of particle size distribution is achievable without the addition of a stabilizing agent (e.g. polyvinyl alcohol, PVA) [13]. Additionally, an excess of chloride ions was shown to facilitate better mixing between precursors leading to a higher degree of alloying and better composition control, resulting in improved activity and selectivity in the oxidation of benzyl alcohol compared to counterpart catalysts prepared by the other two more conventional methods.

Here, we show that by combining Pd and Zn, similar catalytic activity can be obtained to that displayed by the Au–Pd system in the aerobic oxidation of benzyl alcohol. The remarkable activity displayed has been assigned to the formation of PdZn nanoparticles having a mean size of 2.5 nm and a very narrow particle size distribution. The Pd–Zn family of bimetallic catalysts are more traditionally used in methanol steam reforming reactions [14], but here we now show they can also be utilized in selective oxidation reactions.

## Experimental details

### Catalyst preparation

Monometallic and bimetallic catalysts, with 1% total metal loading, supported on TiO_2_ were prepared using a modified impregnation method, which employs an excess of Cl^−^ ions to ensure complete dissolution and effective mixing of the metal precursor salts. For the preparation of mono- and bi-metallic supported catalysts, ZnCl_2_ (98%, Sigma Aldrich) and PdCl_2_ (99%, Sigma Aldrich) were used as the metal precursors. In a typical synthesis sequence, the requisite amount of zinc chloride and/or palladium chloride solution with total HCl concentration of 0.58 M were charged into a clean 50 mL round-bottom flask containing a magnetic stirrer bar. The volume of the solution was adjusted by adding deionized water to obtain a total volume of 16 mL. The flask was then immersed into an oil bath sitting on a magnetic stirrer hot plate. The solution was stirred vigorously at 1000 rpm and the temperature of the oil bath was raised from room temperature to 60 °C over a 10 min time period. Upon reaching 60 °C, 1.98 g of the TiO_2_ support material (Degussa Evonik P25) was gradually added over a period of 10 min with constant stirring. After all the support material was added, the slurry was stirred at 60 °C for an additional 15 min. Following this, the oil bath temperature was raised to 95 °C, and the slurry was stirred overnight until all the water had evaporated leaving a dry solid. Subsequently the solid was ground thoroughly in a mortar and pestle to form a uniform powder. The catalysts were further reduced in a flow of 10% H_2_/Ar for 4 h at either 300, 400 or 500 °C.

### Benzyl alcohol oxidation

Benzyl alcohol oxidation measurements were carried out in a Radleys carousel reactor using a 50 mL glass stirred reactor. In a typical reaction run, the requisite amount of catalyst and substrate were charged into the reactor at room temperature which was then purged three times with O_2_ before sealing the reactor using a Teflon screw threaded cap. The reactor, containing the reaction mixture, was loaded into a pre-heated block, which was maintained at the reaction temperature. The reaction was initiated by commencing stirring inside the reactor with a magnetic bar at 1000 rpm. After a specific time, the stirring was stopped, and the reactor was cooled in an ice bath for 10 min. Following this, the reactor was carefully opened, and the contents were centrifuged. An aliquot of the clear supernatant reaction mixture (0.5 mL) was then diluted with mesitylene (0.5 mL) for GC analysis (Gas Chromatograph: Varian Star 3800CX with a 30 m CP-Wax 52 CB column). The products were identified by comparison of their retention time with known standards. For quantifying of the amount of reactants consumed and products generated, a calibration method using mesitylene as the external standard was employed. It was verified that no reaction occurred in the absence of the PdZn/TiO_2_ catalyst or in the presence of the TiO_2_ support alone. The turnover frequency (TOF) for the reaction was calculated from the moles of substrate consumed and the total number of moles of metal present in the catalyst.

### Catalyst characterization

#### 1.1.1. Surface area measurement (BET)

The Brunauer–Emmett–Teller (BET) method was used with a Quantachrome NOVA e-Series surface area analyser to determine the surface area of the catalyst samples. Initially the sample was degassed under vacuum at 120 °C for 1 h to remove any adsorbed water on the surface. The sample was then cooled to room temperature before releasing the vacuum. The degassed samples were then weighed, and a 5-point surface area analysis was carried out with the samples submerged in liquid nitrogen.

#### 1.1.2. Temperature programmed reduction (TPR)

Temperature programmed reduction analysis was carried out on a Thermo TPD/R/O 1100 series instrument equipped with a thermal conductivity detector (TCD). The catalyst (50 mg) was heated up to 800 °C at 5 °C min^−1^ under an atmosphere of 10% H_2_/Ar (15 ml min^−1^) after pre-treatment at 110 °C in Ar for 45 min.

#### 1.1.3. X-ray photoelectron spectroscopy (XPS)

A Thermo Scientific K-Alpha^+^ photoelectron spectrometer was used to collect XP spectra utilising a micro-focused monochromatic Al K_α_ X-ray source operating at 72 W. Data was collected over an elliptical area of approximately 400 μm^2^ at pass energies of 40 and 150 eV for high-resolution and survey spectra, respectively. Sample charging effects were minimised through a combination of low energy electrons and Ar^+^ ions, consequently this resulted in a C(1s) line at 284.8 eV for all samples. All data was processed using CasaXPS v2.3.20 rev 1.2H using a Shirley background, Scofield sensitivity factors and an energy dependence of −0.6.

#### 1.1.4. Scanning transmission electron microscopy (STEM)

Samples for examination by STEM were prepared by dry dispersing the catalyst powder onto a holey carbon film supported by a 300-mesh copper TEM grid. Bright field (BF) and high angle annular dark field (HAADF) STEM images were taken using an aberration corrected JEM ARM-200CF microscope operating at 200kV. This instrument was also equipped with a JEOL Centurio silicon drift detector for X-ray energy dispersive spectroscopy (XEDS). Particle size distributions were determined from analysis of the HAADF electron micrographs using Image J.

### Computational methods

Periodic plane-wave DFT calculations were performed using the Vienna *Ab initio* Simulation Package (VASP) [15,16], the Perdew–Burke–Ernzerhof functional revised for solids [17] and a kinetic energy of 450 eV to expand the plane-waves of the Kohn-Sham valence states. The inner electrons were represented by the projector-augmented wave (PAW) pseudo-potentials considering also non-spherical contributions from the gradient corrections [18]. All the calculations include the long-range dispersion correction approach by Grimme [19,20], which is an improvement on pure DFT when considering large polarizable atoms [21–26]. The optimisation thresholds were 10^–5^ eV and 0.03 eV/Å for electronic and ionic relaxation, respectively. The Brillouin zone was sampled by Γ-centred k-point mesh generated through a Monkhorst-Pack grid of 5×5×1 K-points, which ensures the electronic and ionic convergence [27]. In order to improve the convergence of the Brillouin-zone integrations, the partial occupancies were determined using the tetrahedron method with Blöch corrections smearing with a set width for all calculations of 0.2 eV. Open shell calculations were tested leading to close shell results.

The Pd bulk lattice parameter is 3.893 Å [28], which is in very good agreement with that resulting from the cell optimization (3.839 Å). The (111) surface was simulated by a slab model containing five atomic layers where the two uppermost layers were relaxed without symmetry restrictions and the bottom ones were frozen at the bulk lattice parameter. The slab contained 45 atoms per unit cell exposing an area of 57.422 Å^2^. We added a vacuum width of 15 Å between periodic slabs, big enough to avoid the interaction between periodic images. The PdZn alloy structures were created by randomly substituting Pd by Zn atoms in different configurations and amounts, as summarised in Table 1. The slab lattice parameters were allowed to vary. The molecules in the gas phase were relaxed in an asymmetric box large enough to avoid any interaction with periodic images. The binding energy (E_B_) was defined as the difference between the isolated species and the combined system. Hence a negative value indicates a favourable interaction. The reaction energy (E_R_) of each step was calculated as the total energy difference between the final state (product(s)) and the initial state (reactant(s)).

**Table 1.** Lattice parameter (a), its variation (Δa) with respect pure Pd, and the atomic arrangement of PdZn alloys with different Zn:Pd ratio in the slab model. The periodic cell is depicted by black lines, with Pd and Zn atoms as grey and green spheres respectively.

Zn:Pd ratio	0:1	0.29:1	1.04:1	2.75:1
% Pd in Zn-Pd slab model (molar %)	100%	78%	49%	26%
a (Å)	3.839	3.799	3.772	3.751
Δa (Å)	0.0	−0.040	−0.067	−0.088
Atomic arrangement				

## Results and discussion

### 1.2. Catalyst testing results

It has been reported previously that each component of the bimetallic catalyst might behave differently in a particular catalytic reaction when studied individually [29]. Therefore, first we experimentally tested a range of bimetallic 1wt% Pd–Zn/TiO_2_ catalysts and their monometallic counterparts for benzyl alcohol oxidation to gain further insight into the influence of each of the elemental components on catalytic activity and selectivity (Table 2).

**Table 2.** Activity and selectivity of the 1wt% (Pd–Zn)/TiO_2_ catalyst in the benzyl alcohol oxidation reaction. Catalysts were reduced at 400 °C in 10%H_2_/Ar for 4h.

Amount of Pd in the Pd–Zn alloy catalyst (molar %)	Conversion (%)	Selectivity (%) (to benzaldehyde)
100	52	75
90	55	81
65	54	71
50	52	67
38	41	79
0	3	88

Reaction conditions: Benzyl alcohol 2 g, catalyst 20 mg, T = 120 °C, p = 1 bar, t = 1 h

As can be seen from Table 2, the monometallic Pd and Zn catalysts exhibited lower activity than any of their bimetallic counterparts. It has been reported previously that a Pd-only catalyst favours the disproportionation reaction, resulting in a higher selectivity to toluene [30]. Addition of Zn to Pd helps to suppress the acidic centres responsible for producing the undesired product. However, by comparing the performance of samples prepared with different proportions of Zn and Pd in the alloy, it is clear that the actual Zn to Pd ratio plays a very important role. The amount of Zn has to be carefully controlled as Zn molar concentrations above 20% have a tendency to decrease the conversion in the reaction. As discussed previously, selectivity in the benzyl alcohol reaction is strongly affected by the two reaction pathways, namely disproportionation and oxidation that dictate the final product distribution. Therefore, it is crucial to prepare alloy nanoparticles with the correct composition to block the undesired disproportionation reaction pathway.

It is well known that the presence of a second metal with Pd under reducing conditions can lead to alloying between the two components. Furthermore, because the precise reduction temperature may have some influence on the degree of metal reduction, as well as the size, structure and composition of the alloy particles, we decided to investigate in some detail how important this parameter is for a 90%Pd-10%Zn/TiO_2_ catalyst.

The activity and selectivity data presented in Table 3 show that by increasing the reduction temperature, a lower overall conversion but a higher selectivity to benzaldehyde was obtained. This might be related to the presence of less monometallic palladium on the catalyst surface upon reduction at a higher temperature, which would be responsible for the disproportionation reaction. In conjunction with this, it is expected that more Zn–Pd particles would be formed at higher reduction temperatures, lowering the overall conversion in the reaction.

**Table 3.** Influence of reduction temperature on the activity of a 90%Pd-10%Zn/TiO_2_ catalyst for benzyl alcohol oxidation.

Reduction temperature (°C)	Conversion (%)	Selectivity to benzaldehyde (%)
300	84.8	63.5
400	55.2	80.7
500	21.8	87.1

Reaction Conditions: Benzyl alcohol 2 g, catalyst 20 mg, T = 120 °C, t = 1 h

### 1.3. Physical and structural characterisation

Physical characterisation of this same set of materials (Table 4) showed that a higher reduction temperature led to lower overall surface area and lower exposed surface area. Additionally, after reduction at higher temperatures, the electronic interaction between Pd and Zn diminished the CO chemisorption capacity, suggesting the presence of more Pd–Zn alloy species. The dispersion of Pd also dropped from 22.0 to 3.2% when the reduction temperature was increased from 300 to 500 °C.

**Table 4.** Physical characterisation of a 90%Pd-10%Zn/TiO_2_ catalyst reduced at different temperatures.

Reduction temperature (°C)	Catalyst surface area (m^2^g^−1^)	Exposed metal surface area (m^2^g^−1^)	Pd dispersion (%)	CO uptake (µmol/g)
300	48.75	1.10	22.00	23.00
400	46.39	0.68	15.71	14.25
500	41.62	0.72	3.24	5.23

#### 1.3.1. Temperature programmed reduction

**Figure 1.**
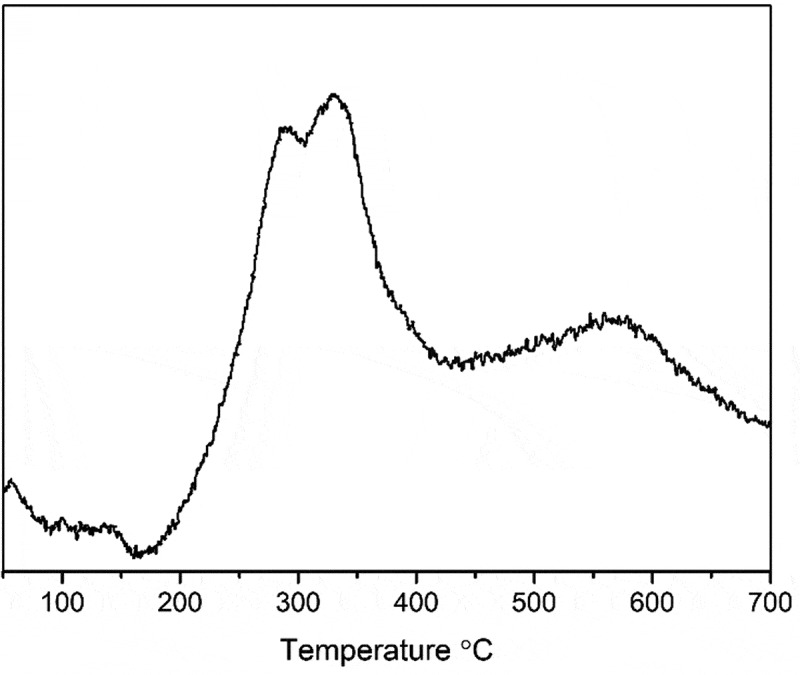
TPR profile for fresh 90%Pd-10%Zn/TiO_2_ catalyst.

Figure 1 shows the temperature programmed reduction (TPR) profile for the fresh, uncalcined 1%PdZn/TiO_2_ material. It is interesting to note the lack of a peak at around ~110 °C which is commonly associated with the reduction of PdO. This might be due to the very low loading of metal in the sample. However, three strong reduction peaks are seen at temperatures of 289, 329 and 577 °C. The peak around 577 °C is ascribed to the reduction of Ti^4+^ ions [31]. The two peaks at lower temperature are believed to be a result of TiO_2_ surface reduction. The addition of Pd and Zn increases the reducibility of the support, causing a decrease in reduction temperature [32]. The TPR profile presented in Figure 1 can help to explain the measured difference in catalytic activity for materials reduced at different temperatures. A reduction treatment at 300 °C would not be sufficient to reduce all the surface TiO_2_ species, so a higher reduction temperature is desirable. It is likely that the peak at 289 °C is associated with Pd-TiO_2_ species, while that at 329 °C is associated with Zn-TiO_2_ entities. Therefore, when a reduction temperature of 400 °C is employed, these species both become reduced, leading to the increase in selectivity to benzaldehyde. This is in good agreement with observations in Table 2, as using a monometallic Pd/TiO_2_ catalyst for benzyl alcohol oxidation results in a relatively low selectivity to benzaldehyde, whereas monometallic Zn/TiO_2_ displays an improved selectivity.

#### 1.3.2. X-ray photoelectron spectroscopy characterization

**Figure 2.**
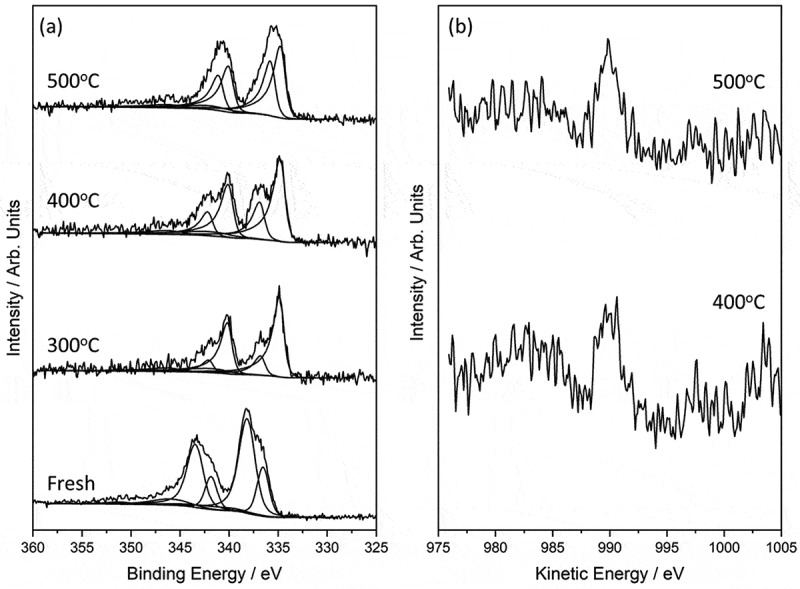
(a) Pd(3d) XPS signal from the unreduced 90%Pd-10%Zn/TiO_2_ catalyst, and the same material reduced at 300, 400 and 500 °C. (b) Difference spectra for the Zn(LMM) Auger peaks for the materials subjected to the 400 and 500 °C reductive treatments.

The core-level photoelectron spectra for the series of 90%Pd-10%Zn/TiO_2_ catalysts reduced at different temperatures are shown in Figure 2(a). The unreduced catalyst consists of two Pd species, with binding energies of 338.1 and 336.5 eV, which we ascribe to palladium chloride and palladium oxide species respectively, the former supported by the detection of a significant amount of chlorine on the surface.

After the reduction treatment, two distinct Pd species are still detected but now with binding energies of 334.8 and 336.7 eV. As the reduction temperature is increased, the concentration of the species at 336.7 eV progressively increases while the concentration of the lower binding energy species decreases; no significant shift in binding energy for either was noted as a function of reduction temperature. The two species here are attributed to a Pd–Zn alloy (336.7 eV) and metallic Pd (334.8 eV). Whilst the binding energy of the alloy is identical to that which we have previously ascribed to a palladium chloride type species, our assignment is strengthened by analysis of the Zn LMM Auger signal in the reduced samples. These reveal a signal with a kinetic energy of 989.8 eV, which accompanied with the Zn(2p_3/2_) photoelectron peak at a binding energy of 1022.1 eV, yields a modified Auger parameter of 2011.9 eV, consistent with the range associated with Zn-alloy species [33]. The difference spectra as revealed by the vector analysis method [34] are shown in Figure 2(b) for the samples subjected to the 400 and 500 °C reduction temperatures.

#### 1.3.3. STEM characterisation

**Figure 3.**
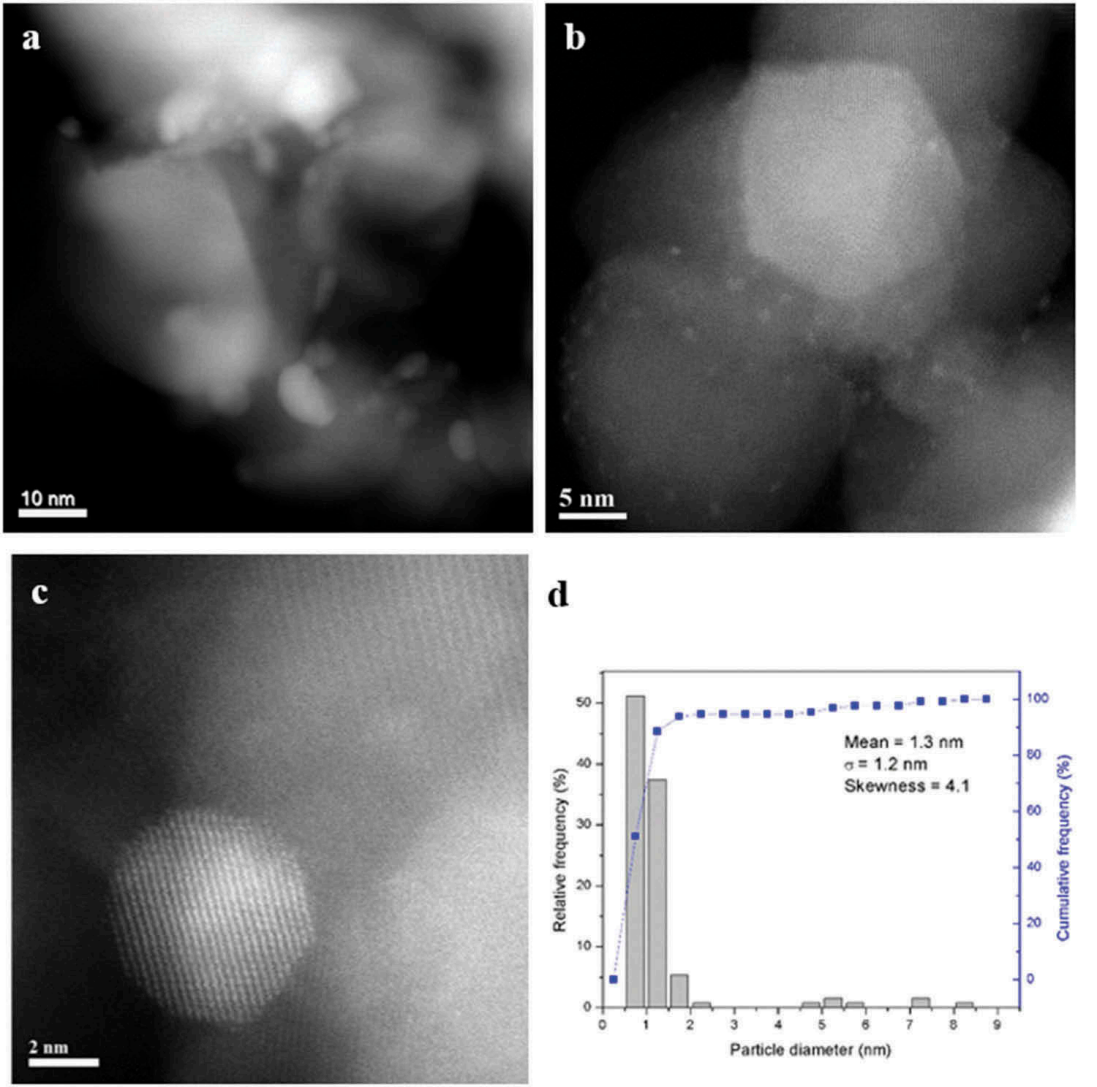
(a–c) Representative HAADF STEM images of the 90%Pd-10%Zn/TiO_2_ sample reduced in 10% H_2_/Ar at 300 °C for 4 h. (d) Corresponding metal particle size distribution from this sample. The lattice fringe structure in (c) is consistent with the [110] projection of an fcc metal.

**Figure 4.**
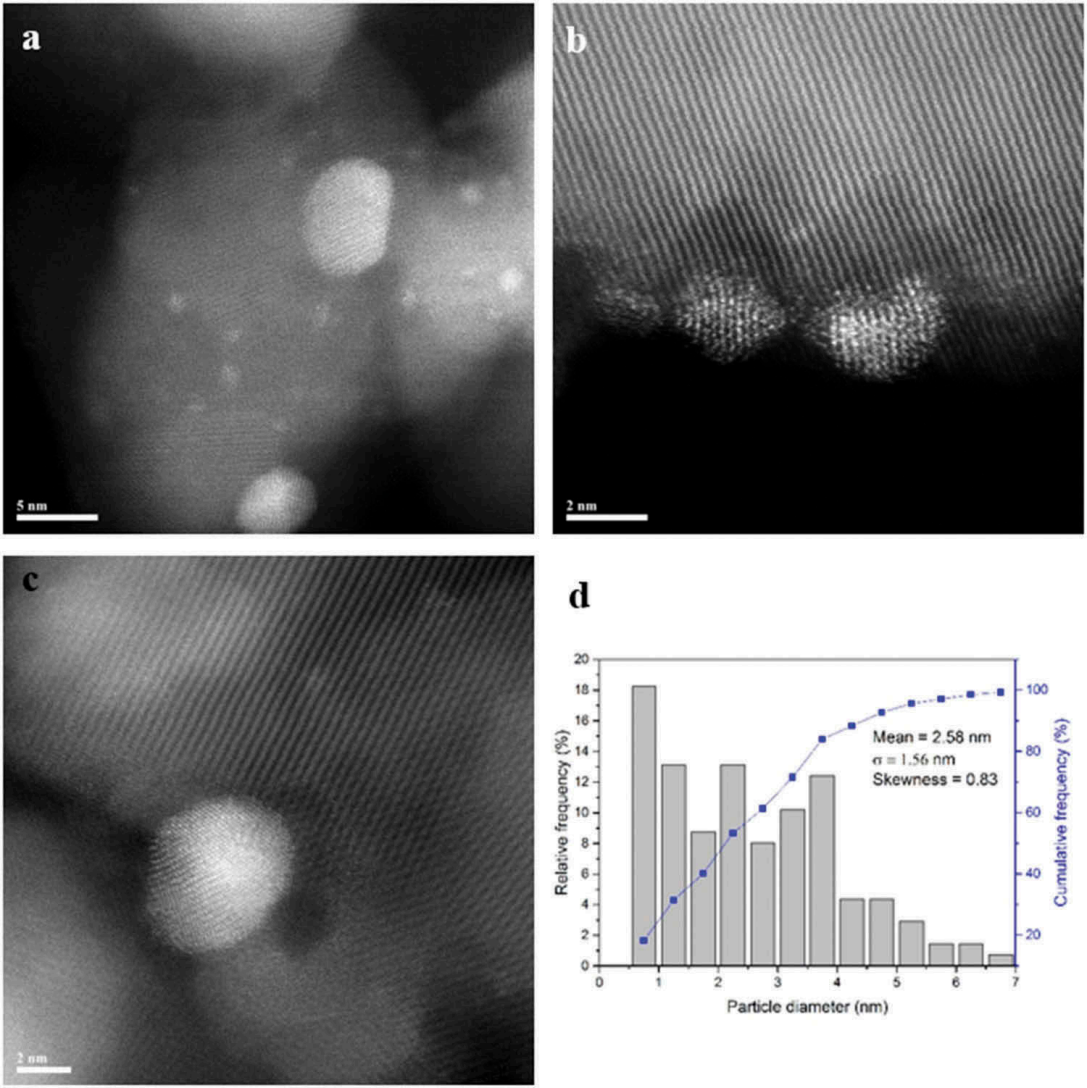
(a–c) Representative HAADF STEM images of the 90%Pd-10%Zn/TiO_2_ sample reduced in 10% H_2_/Ar at 400 °C for 4 h. (d) Corresponding metal particle size distribution from this sample. The lattice fringe structure in (c) is consistent with the [12] projection of the tetragonal Pd–Zn phase.

**Figure 5.**
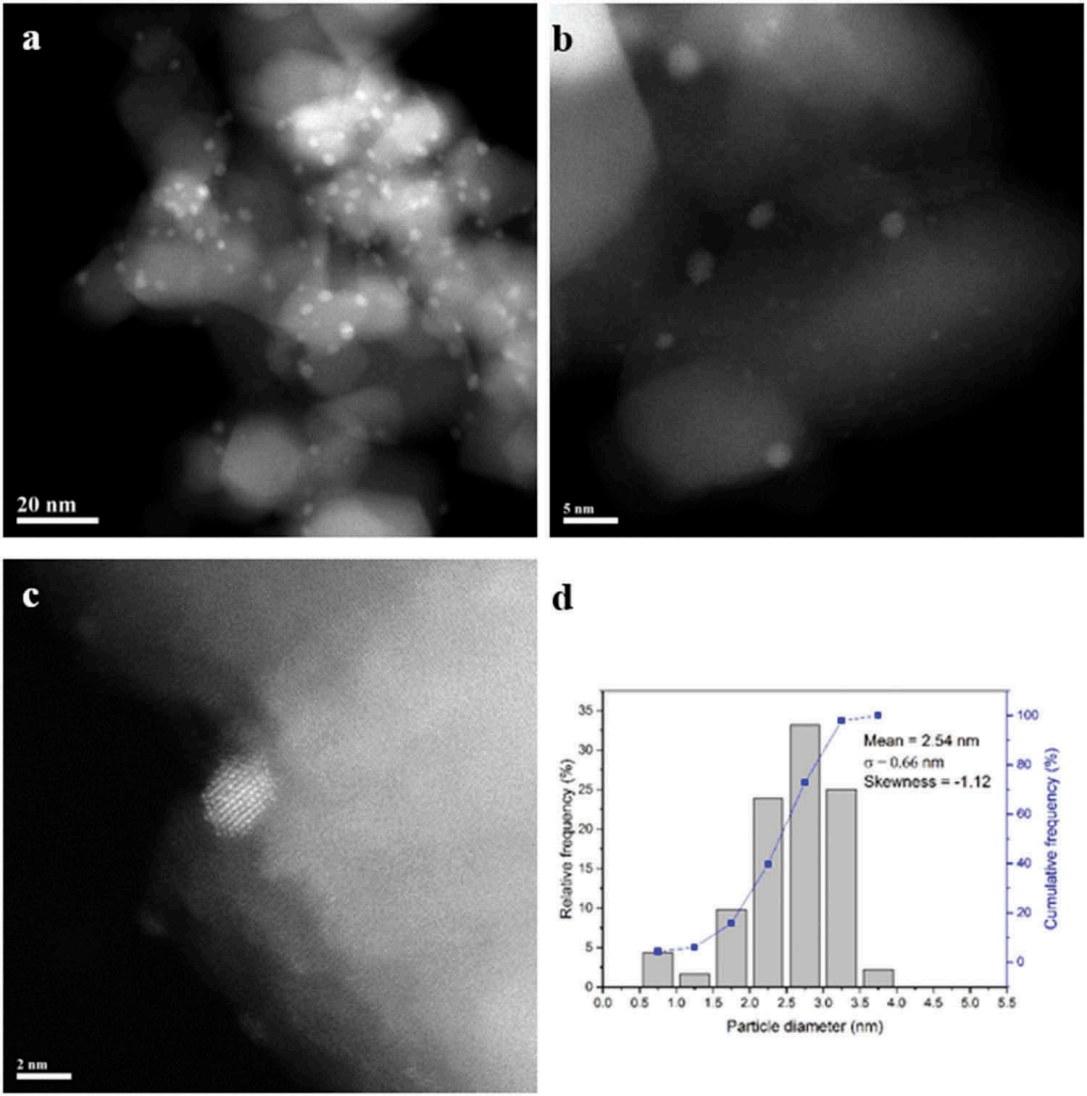
(a–c) Representative HAADF STEM images of the 90%Pd-10%Zn/TiO_2_ sample reduced in 10% H_2_/Ar at 500 °C for 4 h. (d) Corresponding metal particle size distribution from this sample.

The set of 90%Pd-10%Zn/TiO_2_ materials reduced at 300, 400 and 500 °C were also characterised by STEM-HAADF imaging and XEDS analysis. In the 300 °C reduced sample (Figure 3(a)) there is a definite bimodal size distribution of particles (Figure 3(d)) with a large population of poorly crystalline 1–2 nm PdO_x_ particles (Figure 3(b)) and more occasional larger (5–10 nm) crystalline particles (Figure 3(c)). These latter particles typically displayed an fcc (face centred cubic) structure which could be consistent with either (i) pure Pd particles or (ii) Pd particles substitutionally doped with low levels (<9%) of Zn atoms. Upon reduction at 400 °C, this bimodal size population was still found to persist (Figure 4(a,d)), but the smaller particles were now much more crystalline in character. Furthermore the ‘larger’ Pd–Zn particles now had relatively smaller diameters (5–7 nm) as compared to those in the 300 °C reduced sample. Analysis of the interplanar spacing and angles of some of these larger particles often showed them to be consistent with the PdZn tetragonal intermetallic phase (Figure 4(c)). This is the stable structure for Pd–Zn particles containing between 38 and 50% Zn. The presence of Zn in these latter particles was also confirmed by XEDS analysis. When the sample was reduced at 500 °C the dispersed metal particles showed a much more uniform size distribution in the 1–4 nm range (Figure 5(a,b,d)) and a high degree of crystallinity (Figure 5(c)). These supported metal particles were confirmed by XEDS to contain both Pd and Zn. Most of the nanoparticles were consistent with the dilute Zn-doped Pd fcc phase, although some occasional Pd–Zn tetragonal phase particles were also detected.

#### 1.3.4. Computational calculations

Computational calculations were applied to estimate the most beneficial composition of Pd–Zn nanoparticles for the oxidation of benzyl alcohol. The adsorption of reactants and products on both monometallic Pd and Pd–Zn alloys with a Pd/Zn ratio between to 2.75 to 0.29 was modelled. This resulted in 129 non-equivalent arrangements of the molecules on the surfaces. The binding energies depend on the amount of Zn in the nanoparticles, hence, modifying the stabilisation of intermediates along the reaction mechanism. Binding energies, including deviations of other configurations, and selected geometrical descriptors of adsorbed molecules in its most stable configuration are summarised in Table 5.

**Table 5.** Reactant and product binding energies (E_B_) and average values of distances (d) of the aromatic ring from the pure Pd and Pd–Zn alloy (111) surfaces.

Composition of Pd–Zn alloy (molar %)		E_B_ (eV)	d_surf-ring_ (Å)
100% Pd	Benzyl alcohol	−3.40 ± 0.00	2.067
	Benzaldehyde	−2.66 ± 0.12	2.022
	Benzoic acid	−3.23 ± 0.00	2.075
	Toluene	−2.97 ± 0.01	2.073
78%Pd-22%Zn	Benzyl alcohol	−3.05 ± 0.32	2.146
	Benzaldehyde	−3.14 ± 0.24	2.083
	Benzoic acid	−2.73 ± 0.25	2.123
	Toluene	−2.59 ± 0.27	2.143
49%Pd-51%Zn	Benzyl alcohol	−1.72 ± 0.06	2.594
	Benzaldehyde	−1.79 ± 0.17	2.282
	Benzoic acid	−1.55 ± 0.10	2.287
	Toluene	−1.42 ± 0.07	2.318
26%Pd-74%Zn	Benzyl alcohol	−1.35 ± 0.04	2.957
	Benzaldehyde	−1.33 ± 0.06	2.443
	Benzoic acid	−1.11 ± 0.05	3.102
	Toluene	−1.04 ± 0.03	2.961

**Figure 6.**
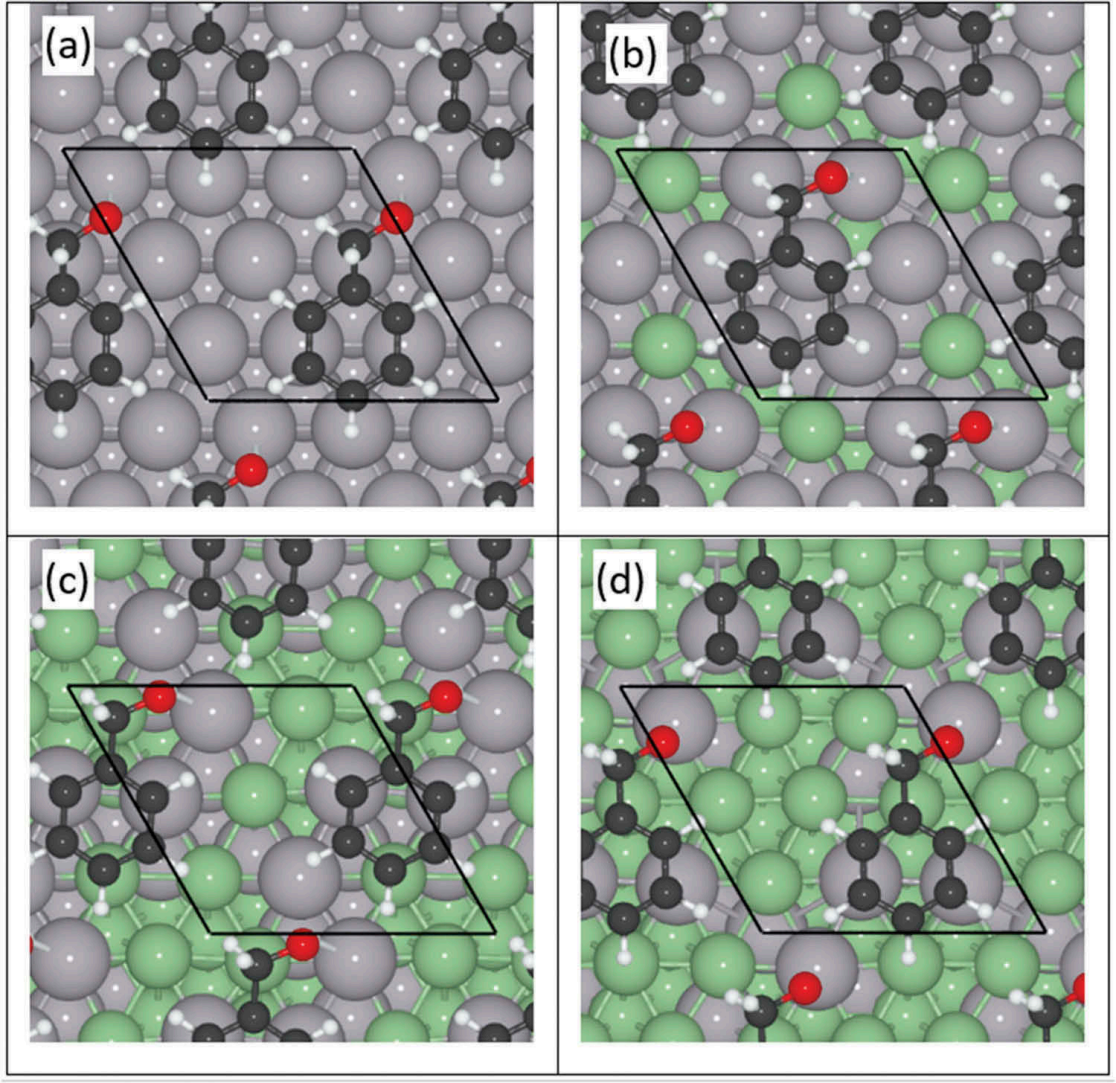
Top view representation of benzyl alcohol adsorbed on Pd and various PdZn alloy (111) surfaces: (a) 100% Pd, (b) 78%Pd-22%Zn, (c) 49%Pd-51% Zn and (d) 26%Pd-74%Zn. The periodic cell is depicted by black lines; Pd and Zn atoms are represented by grey and green spheres respectively.

The values listed in Table 5 relate the amount of Zn present in the alloy system with the binding energies of the benzyl alcohol molecule. It shows a progressively weaker interaction between the reactant molecule and the surface with increasing concentration of Zn as well as lower distortion of the aromatic ring plane, i.e. the hydrogen atoms moving out of the phenyl plane. Figure 6 schematically represents the most stable configurations of the benzyl alcohol molecule on the different surfaces. These illustrations show the preference of the molecule to lay on Pd rather than on Zn regions, while the hydroxyl proton always points towards the surface in a practically perpendicular manner.

**Figure 7.**
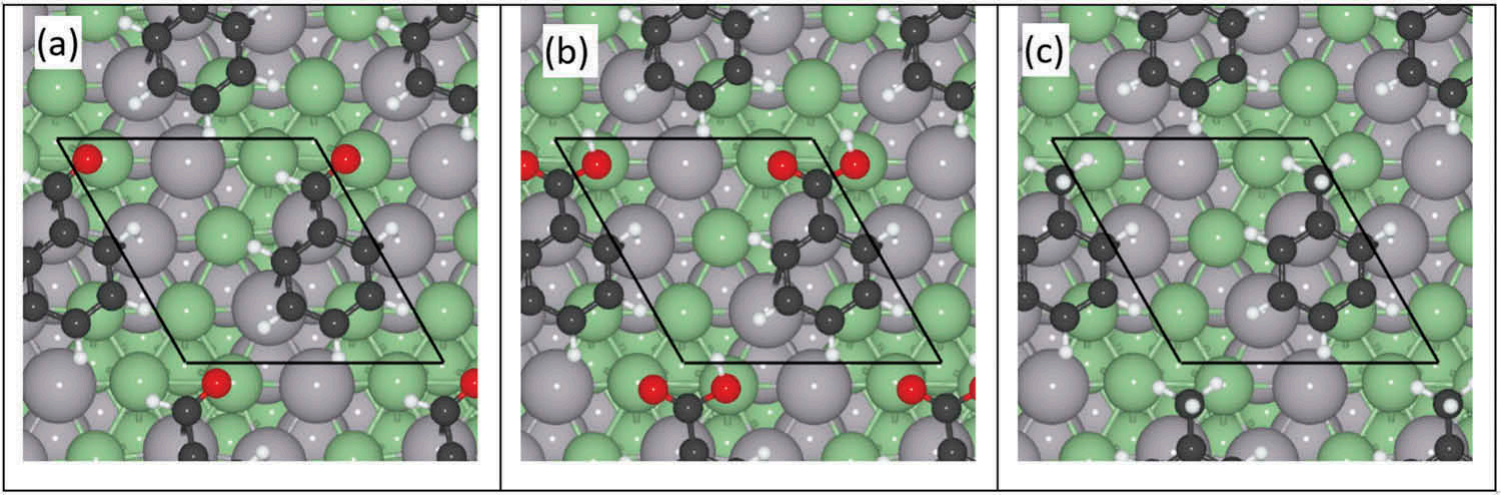
Top view representation of (a) benzaldehyde, (b) benzoic acid, and (c) toluene on the surface of a 49%Pd-51%Zn alloy. The periodic cell is depicted by black lines; Pd and Zn atoms are represented by grey and green spheres respectively.

The interaction of the possible reaction products from benzoic acid (i.e. benzaldehyde, benzoic acid and toluene) with the surfaces of different compositions is also summarised in Table 5 and depicted schematically in Figure 7 for the 49%Pd-51%Zn alloy. While from Figure 7 we can see the same preference to maximize the interaction of the adsorbate molecule with the surface Pd metal atoms, the binding energies listed in Table 5 suggest the stabilisation of benzaldehyde on the surface in the presence of Zn, whereas the opposite trend is true for benzoic acid and toluene. The magnitude of these binding energies also implies that the surface with low Zn loading will be maximally covered by these molecules.

**Figure 8.**
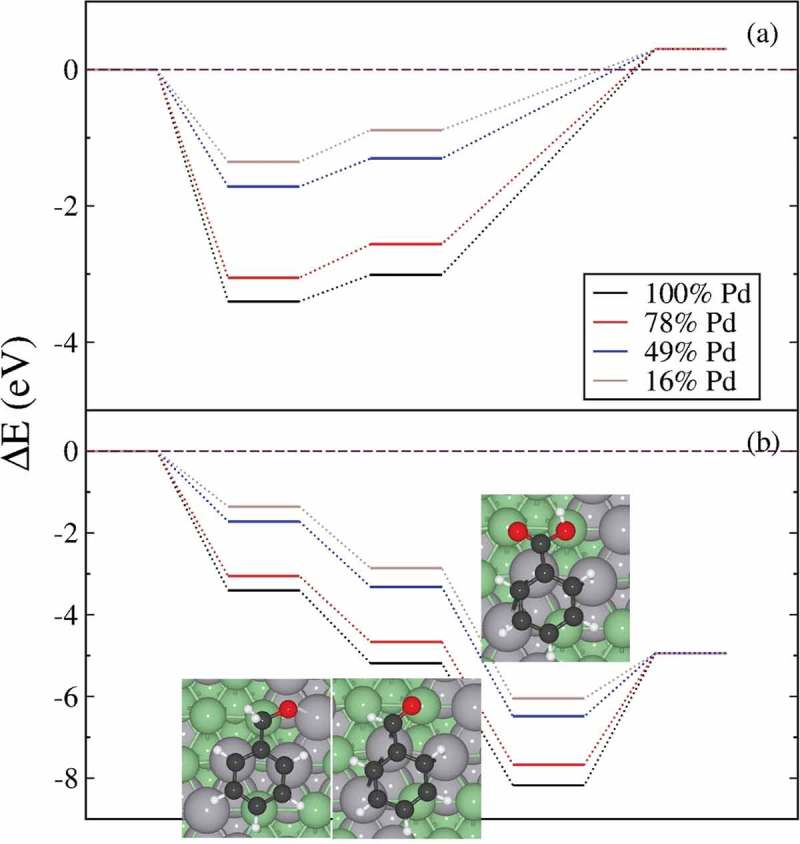
Thermodynamic profile for (a) the non-oxidative disproportionation and (b) the oxidative reaction pathway. The profiles follow as: adsorption of benzyl alcohol on the naked surface, conversion to benzaldehyde and benzoic acid before desorbing from the surface. We have considered the energy of H_2_O and O_2_ molecules according to the reaction pathways.

We have expressed, in Figure 8, the different system energies as a function of isolated benzyl alcohol and naked surfaces according to reactants in the non-oxidative (disproportionation) and, adding isolated oxygen, in the oxidative pathway presented in Equations 1 and 2. Thus, Figure 8 shows that the production of benzaldehyde and toluene via benzyl alcohol disproportionation is energetically unfavourable, i.e. endothermic, although their formation and desorption is more likely on systems with higher loadings of Zn. On the other hand, the oxidative process is exothermic, and the alcohol will be oxidized to aldehyde and later to acid in the presence of molecular oxygen. The adsorption of benzyl alcohol is less exothermic on surfaces with higher Zn content but, still, the oxidation reaction drives the process. The desorption of benzoic acid is slightly endothermic, but the energy of the oxidative products in the gas phase still remains below the one of reactants, indicating a favourable process.

## 2. Discussion

Computational calculations showed that the benzyl alcohol molecule has more stable configurations on Pd rather than on Zn atoms, which can explain higher conversion in the overall reactions, when comparing catalysts with various Pd to Zn ratios. Additionally, it was found that the disproportionation reaction is more likely to take place on the Pd rich surfaces, which is in agreement with previous experimental findings. Further it shows that the stabilization of benzaldehyde is more likely to take place on Zn, rather than Pd atoms, and illustrates the importance of some Zn metal also being present in the nanoparticle. The computational calculations seem to be in general agreement with data presented in Table 2. We consider that Pd is the metal component primarily responsible for high activity of the catalyst, while role of the Zn is more limited and related to reaction selectivity. However, the ratio between the two metals needs to be carefully controlled as an excess amount of Zn may result in the formation of Zn-rich alloy nanoparticles, leading to a drop in the level of conversion.

Optimization of the catalyst preparation method is another vital aspect to consider for reaching high catalytic activity. The material calcined at temperatures ≥ 400 °C exhibited very good selectivity, compared to catalysts calcined at 300 °C. It is clear that the higher temperature calcination treatments significantly influenced the size distribution of the nanoparticles and ensured the removal of any non-alloyed Pd or PdO_x_ particles. It seems desirable to generate fully alloyed Pd–Zn nanoparticles in the 2.5–2.8 nm size range to have a highly active Pd–Zn/TiO_2_ catalyst that displays high selectivity to benzaldehyde. In contrast with the materials just calcined at 300 °C, the samples reduced at higher temperatures contained no larger particles (>7 nm) which may have been associated with low selectivity in benzyl alcohol oxidation. Additionally, as confirmed by XPS and STEM analyses, the sample calcined at 300 °C contained monometallic Pd or PdO_x_ particles that are associated with higher activity but a lower selectivity to benzaldehyde. The noticeable drop in conversion observed for the Pd–Zn/TiO_2_ material calcined at 500 °C is most likely associated with low Pd dispersion as measured by CO chemisorption analysis. As the analysis focuses on CO uptake on available Pd sites, those must have been significantly reduced in number upon calcination, resulting in exposure of relatively more Zn sites. The catalyst calcined at 400 °C contained more PdZn intermetallic nanoparticles exhibiting the tetragonal phase, as compared to the catalyst calcined at 500 °C which predominantly showed Pd–Zn nanoparticles having an fcc structure with a much more dilute amount of Zn present in solid solution.

As discussed earlier, catalysts comprising Pd–Zn nanoparticles supported on TiO_2_ are not normally associated with oxidation reactions. Our work shows that by carefully optimizing the Pd–Zn/TiO_2_ catalyst nanostructure and composition, it is possible to achieve similar catalytic performance results to previously studied 1%Au–Pd/TiO_2_ catalysts. Sankar et al. reported that a 1%Au–Pd/TiO_2_ catalyst, prepared by the modified impregnation method (calcined at 400 °C) and tested under identical conditions, gave a 56% conversion with 74% selectivity to benzaldehyde [13]. Our 1%Pd–Zn/TiO_2_ catalyst displayed an activity of 55% with 81% selectivity to benzaldehyde. This demonstrates that zinc could be a suitable and cheaper replacement for gold in Pd-based bimetallic catalysts designed for the oxidation of benzyl alcohol to benzaldehyde.

## Conclusions

The present work shows that the selective oxidation of benzyl alcohol to benzaldehyde can be achieved with a Pd–Zn/TiO_2_ catalyst. It was found that the optimal composition of the alloy nanoparticles contain 90% Pd and 10% Zn. Computational calculations confirmed the experimental findings, predicting the stabilization of benzyl alcohol on Pd sites, and benzaldehyde on Zn sites, respectively. The reduction pre-treatment of supported Pd–Zn/TiO_2_ catalysts exerted a strong effect on catalyst selectivity with material reduced at above 400 °C giving the best catalytic performance results. The origin of high selectivity lies in generating an appropriate narrow size distribution of 2–4 nm PdZn alloy nanoparticles containing a dilute amount of Zn.
